# Quality of life and Magnetic resonnance imaging(MRI) in multiple sclerosis

**DOI:** 10.1192/j.eurpsy.2025.729

**Published:** 2025-08-26

**Authors:** N. Ben Hamed, S. Cherif, A. Aissa, A. Souissi, R. Jomli

**Affiliations:** 1 psychiatry A; 2 Psychiatrt A, Razi hospital; 3 neurology department, LR18SP03 and Clinical Investigation Center Neurosciences and Mental Health - university hospital center Razi, Manouba, Tunisia

## Abstract

**Introduction:**

Multiple sclerosis (MS) is a chronic neurological disorder that can profoundly affect patients’ quality of life due to its diverse symptoms and disease progression. MRI findings are critical in assessing the extent of neurological damage and may correlate with various aspects of patients’ health and well-being.

**Objectives:**

identify the relationship between MRI findings and quality of life in individuals with MS.

**Methods:**

A cross-sectional study was conducted in the neurology department of Razi University Hospital (Tunisia) between October 2023 and June 2024. Patients with a diagnosis of MS based on the 2017 McDonald criteria were recruited, excluding those with active disease relapses. Participants completed questionnaires covering sociodemographic data, medical history, disability status, clinical and radiological characteristics. Quality of life was assessed using the SF-36 and the Multiple Sclerosis Quality of Life (MSQoL-54) scales.

**Results:**

A total of 83 patients with MS were recruited, with ages ranging from 19 to 66 years. The study population had a predominantly female sex ratio of 3.4. The majority of participants (75.9%) were from urban areas, and 74.7% had a university-level education. Moreover, 49.1% were married, and 60.2% were employed. Regarding medical history, 40.3% had a comorbid condition, and 30.1% had a psychiatric history. The mean age at disease onset was 26 ± 10 years.First-line treatments were prescribed to 27.7% of patients. Second-line treatments were prescribed to 69.9% of patients.

The mean overall score on the SF-36 scale was 53.56. According to MSQ ol54, the most affected domains were sexual function, physical health limitations, and sleep. The least affected domains were pain, social function, and sexual satisfaction.The SF-36 mean score decreased from 50.59 in patients with more than 10 lesions to 21.37 in those with fewer than 10 lesions, with a statistically significant difference (p = 0.012). We found a significant relationship between a higher number of lesions (>10) and the dimensions of physical health (p = 0.007), emotional well-being (p = 0.027), perception of health (p = 0.025), social function (p = 0.017), distress (p = 0.049), cognitive function (p = 0.038), and overall quality of life (p = 0.029). Thalamic lesions were associated with the dimension of distress (p = 0.048). The presence of new lesions in the most recent follow-up MRI was associated with emotional well-being (p=0.018).

**Conclusions:**

The study highlights a significant correlation between MRI findings and quality of life inpatients with MS. Understanding this relationship is crucial for developing targeted treatment strategies that address both the physical and psychological aspects of the disease. By integrating MRI assessments into routine care, healthcare providers can better support the well-being of individuals living with MS and tailor interventions to their specific needs.

**Disclosure of Interest:**

None Declared

